# Biomarker Categorization in Transcriptomic Meta-Analysis by Concordant Patterns With Application to Pan-Cancer Studies

**DOI:** 10.3389/fgene.2021.651546

**Published:** 2021-07-02

**Authors:** Zhenyao Ye, Hongjie Ke, Shuo Chen, Raul Cruz-Cano, Xin He, Jing Zhang, Joanne Dorgan, Donald K. Milton, Tianzhou Ma

**Affiliations:** ^1^Department of Epidemiology and Biostatistics, School of Public Health, University of Maryland, College Park, College Park, MD, United States; ^2^Department of Epidemiology and Public Health, School of Medicine, University of Maryland, Baltimore, Baltimore, MD, United States; ^3^Maryland Institute for Applied Environmental Health, School of Public Health, University of Maryland, College Park, College Park, MD, United States

**Keywords:** biomarker categorization, differential expression, meta-analysis, pan-cancer, transcriptomics

## Abstract

With the increasing availability and dropping cost of high-throughput technology in recent years, many-omics datasets have accumulated in the public domain. Combining multiple transcriptomic studies on related hypothesis via meta-analysis can improve statistical power and reproducibility over single studies. For differential expression (DE) analysis, biomarker categorization by DE pattern across studies is a natural but critical task following biomarker detection to help explain between study heterogeneity and classify biomarkers into categories with potentially related functionality. In this paper, we propose a novel meta-analysis method to categorize biomarkers by simultaneously considering the concordant pattern and the biological and statistical significance across studies. Biomarkers with the same DE pattern can be analyzed together in downstream pathway enrichment analysis. In the presence of different types of transcripts (e.g., mRNA, miRNA, and lncRNA, etc.), integrative analysis including miRNA/lncRNA target enrichment analysis and miRNA-mRNA and lncRNA-mRNA causal regulatory network analysis can be conducted jointly on all the transcripts of the same category. We applied our method to two Pan-cancer transcriptomic study examples with single or multiple types of transcripts available. Targeted downstream analysis identified categories of biomarkers with unique functionality and regulatory relationships that motivate new hypothesis in Pan-cancer analysis.

## Introduction

The revolutionary advancement of high-throughput technology in recent years has generated large amounts of omics data of various kinds (e.g., genetics variants, gene expression and DNA methylation, etc.), which improves our understanding of human disease and enables the development of more effective therapies in personalized medicine (Richardson et al., 2016). As more studies are conducted on a related hypothesis, meta-analysis, by combining evidence from multiple studies, has become a popular choice in genomic research to improve upon the power, accuracy, and reproducibility of individual studies (Ramasamy et al., 2008; Begum et al., 2012; Tseng et al., 2012). One of the main purposes of transcriptomics studies is to identify genes or RNAs that express differently between two or more conditions (e.g., diseased patients vs. healthy controls), also known as differential expression (DE) analysis or candidate biomarker detection. Many meta-analysis methods have been developed or applied to DE analysis, including combining *p*-values (Fisher, 1992) or effect sizes (Choi et al., 2003) and rank-based approaches (Hong et al., 2006). One may refer to Tseng et al. (2012) for an overview of the major meta-analysis methods in transcriptomic studies and Ma et al. (2019) for an overview of available software tools. Yet, a majority of conventional meta-analysis methods only generate a list of differentially expressed genes with strong aggregated evidence without further investigating in what studies are the genes differentially expressed.

Study or population heterogeneity always exists and has been critical to biomarker detection (Di Camillo et al., 2012). For example, The Cancer Genome Atlas (TCGA) consortium completed a Pan-Cancer Atlas of multi-platform molecular profiles spanning 33 cancer types in an effort to provide insights into the commonalities and differences across tumor lineages (Weinstein et al., 2013; Hoadley et al., 2018). When meta-analysis is performed on Pan-cancer transcriptomic studies, we expect to see both DE genes common in all tumor types as well as genes differentially expressed in some tumor types but not others. Biomarker categorization according to their DE patterns across studies is demanding in genomic studies for three reasons. First, biomarkers that share unique cross-study DE patterns are potentially involved in related functions (Berger et al., 2018). Such unique categories of genes with similar function can be used to generate new biological hypotheses. Second, biomarker categorization can make high dimensional genomic data more tractable. For example, in cancer transcriptomic studies, which frequently detect thousands of DE genes, downstream analysis methods such as pathway enrichment analysis or network analysis cannot be applied directly. By partitioning the original large set of DE genes into smaller subsets, biomarker categorization facilitates more focused downstream analysis. Third, RNA sequencing (RNA-seq) technology has led to an explosion of transcriptomic studies profiling both coding (i.e., mRNA) and noncoding RNAs (i.e., miRNA, rRNA, lncRNA, etc.) (Di Bella et al., 2020). Joint analysis of different RNA types with the same cross-study DE patterns can improve understanding of their regulatory relationships, which may lead to inferences about the underlying mechanisms of complex human diseases like cancer.

Li and Tseng (2011) first proposed an adaptively weighted Fisher (AW-Fisher) method for biomarker categorization that assigns a binary weight of 0 or 1 to each study and searches for the pattern of weights that minimizes the aggregate statistics for each gene. Though the method incorporates statistical significance by combining two-sided *p*-values across studies, it does not take into account the direction of regulation (e.g., up-regulated or down-regulated). Other methods incorporate biomarker categorization within the Bayesian framework and combine one-sided *p*-values or Bayesian posterior probabilities (Ma et al., 2017; Huo et al., 2019) but not the magnitudes of effect sizes. In practice, biological significance (i.e., large effect size) and statistical significance (i.e., small *p*-value) do not always occur in tandem (depending on sample size and variance) though they are equally important in interpreting study results (Sullivan and Feinn, 2012; Solla et al., 2018).

**FIGURE 1:**
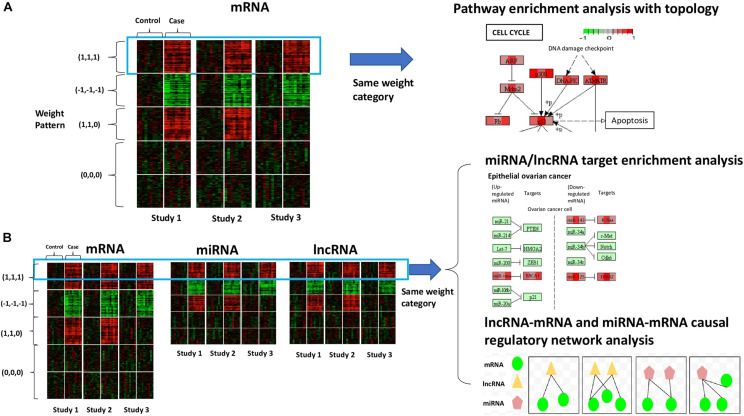
Conceptual framework of our method. **(A)** The scenario with mRNA (or coding genes) only. The heatmap shows the gene expression of all samples from three studies. Rows refer to genes sorted by the specified weight category, columns refer to samples, and solid white lines are used to separate different conditions (control vs. case). Colors of the cells correspond to scaled expression level. The green/red indicates lower/higher expression. Pathway enrichment analysis is applied to genes belonging to the same weight category with topological information to visualize the cross-study DE patterns at the molecular level. **(B)** The scenario with diverse RNA species (e.g., mRNA, miRNA, and lncRNA). The three heatmaps show the expression of different types of transcripts of all samples from three studies, sorted by weight category. In the presence of multiple types of RNA species, we will perform integrative analysis on all the transcripts belonging to the same weight category together. Possible downstream analysis includes miRNA/lncRNA target enrichment analysis and lncRNA-mRNA and miRNA-mRNA causal regulatory network analysis.

In this paper, we propose a novel meta-analysis method to detect and categorize biomarkers by simultaneously considering concordant pattern (i.e., direction of regulation), biological and statistical significance across studies. In addition, we develop a permutation test to assess the uncertainty of the proposed statistics and to control the false discovery rate (FDR). When only coding genes are included, after categorization we perform downstream pathway enrichment analysis with topological information on each category of genes for more biological insights (Figure 1A). In the presence of diverse RNAs, we jointly analyze all RNA species in the same category using miRNA/lncRNA target enrichment analysis and lncRNA-mRNA and miRNA-mRNA causal regulatory network analysis (Figure 1B). We show by simulation that our method detects both concordant and discordant biomarkers and assigns the correct weights. We apply our method to two Pan-cancer transcriptomic data examples: (1) Pan Gynecologic cancer (Pan-Gyn) data with coding genes only; (2) Pan Kidney cancer (Pan-Kidney) data that include mRNA, miRNA as well as lncRNA. The identified biomarker categories show unique functionality and informative regulatory relationships and could suggest new hypotheses about mechanisms underlying exclusive and shared features of different cancer types.

## Materials and Methods

### Popular Meta-Analysis Methods

Tseng et al. (2012) reviewed the major types of meta-analysis methods for DE gene detection in microarrays and classified the methods into four main classes: combining *p*-values, combining effect sizes, combining ranks, and direct merging. We will discuss selected meta-analysis methods from the first two classes that are relevant to our proposed method.

#### Combining *P*-Values

##### Fisher’s method (Fisher, 1992)

The conventional Fisher’s method combines log transformed *p*-value from each study with the statistic $T_{\text{Fisher}}=-2\sum_{k=1}^{\text{K}}\log\left(p_{\text{k}}\right),$ which follows a χ^2^ distribution with 2*K* degrees of freedom under the null hypothesis (i.e., genes not differentially expressed in all studies), where *K* is the number of studies and *p*_k_ is the *p*-value of study *k*, 1 ≤ *k* ≤ *K*.

##### Stouffer’s method (Stouffer, 1949)

The Stouffer’s method proposes inverse normal transformation of *p*-value with the statistic $T_{\text{Stouffer}}\sum_{k=1}^{K}\Phi^{-1}\left(1-p_{k}\right)/\sqrt{K},$ which follows a standard normal distribution under the null, where Φ^−1^(*x*) is the inverse cumulative distribution function of the standard normal distribution.

##### Adaptively weighted fisher’s method (AW-Fisher) (Li and Tseng, 2011)

Fisher’s method does not differentiate DE in a single study or multiple studies as long as their aggregate contribution to the final statistics remains the same. To overcome this and better explain the between study heterogeneity, Li and Tseng (2011) introduced an AW-Fisher’s method as a modification of the original Fisher’s method. The AW-Fisher method considers $U\left(\vec{w}\right)=-2\sum_{k=1}^{K}w_{k}log\left(p_{k}\right)$ for each gene, where $\vec{w}=\left(w_{1},\ldots ,w_{K}\right)$ and each *w_k_* is a binary weight of 0 or 1 assigned to each study k. Denote by $p\left(U\left(\vec{w}\right)\right)$ the *p*-value when the weight $\vec{w}$ is given, the AW-Fisher statistic is defined as: $T_{AW}=\min_{\vec{w}}p\left(U\left(\vec{w}\right)\right)$, where the optimal weight $\left(\hat{w_{1}},\ldots ,\hat{w_{K}}\right)$ that minimizes the *p*-value indicates the subset of studies that contribute to the aggregate statistics and naturally categorizes the biomarkers. There is no closed-form distribution for AW-Fisher statistics under the null, so permutation tests and importance sampling is used to obtain the *p*-value and control the FDR.

#### Combining Effect Size

##### Fixed effect model (FEM) and random effect model (REM) (Choi et al., 2003)

Fixed effect model (FEM) combines effect sizes across all studies for each gene using a simple liner model: $T_{k}=\mu +\varepsilon_{k},\varepsilon_{k}∼N\left(0,s_{k}^{2}\right)$, where μ is the overall mean and the within-study variance $s_{k}^{2}$ represents the sampling error conditioned on study k. The combined point estimate of μ is a weighted average of study-specific effect sizes, where weights are equal to the inverse of $s_{k}^{2}$. FEM will prioritize concordant genes with the same directionality across all studies.

When strong between studies heterogeneity exists and the underlying population effect size is assumed to be unequal across studies, an REM is given hierarchically as $T_{k}=\theta_{k}+\varepsilon_{k},\varepsilon_{k}∼N\left(0,s_{k}^{2}\right);\theta_{k}=\mu +\delta_{k},\delta_{k}∼N\left(0,\tau^{2}\right),$ where between-study variance τ^2^ represents the additional source of variability between studies. A homogeneity test can be performed to test whether τ^2^ is zero or not, and determine the appropriateness of FEM or REM. Like FEM, REM also prioritizes concordant genes but with more flexibility across studies. Neither of FEM nor REM produces biomarker categorization results.

### Remarks

*P*-value combination methods are powerful for detecting genes that have non-zero effects in at least one study (HS_B_ alternative hypothesis setting as in Chang et al. (2013) without considering the magnitudes and directionality of effects across studies. Thus, *p*-value methods cannot distinguish concordant genes (i.e., upregulated or downregulated in all studies) from discordant genes (i.e., upregulated in some studies but downregulated in others). In contrast, effect size combination methods take directionality into account but favor only concordant genes. Even so, discordant genes can still be of interest in, for example Pan-cancer analysis, to understand between tumor heterogeneity. We, therefore, propose a new meta-analysis method that incorporates both *p*-value and effect size combination methods, and considers concordant pattern as well as biological and statistical significance simultaneously to assist biomarker detection and categorization. Here we will introduce our method namely BCMC (**B**iomarker **C**ategorization in **M**eta-analysis by **C**oncordance).

### New Meta-Analysis Method for Biomarker Detection and Categorization

Suppose there are K transcriptomic studies, each study *k* (1 ≤ *k* ≤ *K*) measures the gene expression of *n_k_* samples and *G* genes. We use gene expression as example to introduce our method though the method is ready to analyze other types of transcripts such as miRNA and lncRNA. Our objective in meta-analysis is to detect candidate genes differentially expressed between the case (e.g., patients diagnosed with disease) and control (e.g., healthy subjects) group in multiple studies and categorize the detected genes by their DE patterns across studies. We first perform DE analysis using popular methods such as limma (Ritchie et al., 2015) for microarray or DESeq2 (Love et al., 2014) for RNA-seq in each study and obtain the summary statistics including effect size estimates (log2 fold change or *LFC*_gk_) and *p*-values (*p*_gk_) for each gene *g* (1 ≤ *g* ≤ *G*) in each study *k*. Effect sizes and *p*-values represent biological and statistical significance, respectively, and can be treated as DE evidence for single studies. The smaller the *p*-value and the larger the magnitude of effect size, the more likely a gene will be a DE gene in the study. In meta-analysis, concordance (i.e., a gene having the same sign of effect size in different studies) is regarded as additional piece of DE evidence. We define *g*th gene as being up-regulated in *k*th study when LFC*_gk_* > 0 (i.e., having higher expression in case group) and being down-regulated when LFC*_gk_* < 0 (i.e., having higher expression in control group).

When integrating multiple transcriptomic studies, DE genes may be altered in study-specific patterns. For example, some genes are differentially expressed in all studies while others are only differentially expressed in specific subset of studies. Meta-analysis methods also have different groups of targeted biomarkers as reflected by different statistical hypothesis settings. The null hypothesis for each gene in meta-analysis is commonly defined as: *H*_0_: θ_*g1*_ = ⋯ = θ_gK_ = 0, where θ_gk_ represents the true effect of gene g in study k. Depending on the types of targeted biomarkers, three alternative hypotheses have been proposed in the meta-analysis literature (Birnbaum, 1954; Tseng et al., 2012; Song and Tseng, 2014). The first setting (HS_*A*_) aims to detect DE genes that have non-zero effect in all studies, i.e., θ_gk_ ≠ 0 for all k. The second setting (HS_B_) aims to detect DE genes that have non-zero effect in at least one study, i.e., θ_gk_ ≠ 0 for some k. The third setting (HS_*r*_) aims to detect DE genes that have non-zero effect in at least r studies, i.e., $\sum_{k=1}^{K}I\left\{\theta_{\text{gk}}\neq 0\right\}\geq r$. As we show next, our method generally follows HS_*r*_ setting with specifically *r* = 2 (i.e., we detect DE genes that have non-zero effect in at least two studies).

To detect DE genes and categorize them by cross-study DE patterns, we propose the following two aggregate statistics for each gene that combines DE evidence across up-regulated studies or down-regulated studies, respectively:

$$T_{g\left({\vec{w}}_{g}^{+}\right)}^{+}=\frac{\begin{array}{c} \sum_{LFC_{\text{gk}}>0;LFC_{{\mathrm{gk}}^{\prime }}>0;k\neq k^{\prime }}(w_{\text{gk}}^{+}w_{{\mathrm{gk}}^{\prime }}^{+}LFC_{\text{gk}}LFC_{{\mathrm{gk}}^{\prime }}\\ \left|log_{10}p_{\text{gk}}+log_{10}p_{{\mathrm{gk}}^{\prime }}|\right) \end{array}}{\sum_{k}w_{\text{gk}}^{+}}$$

Tg(w→g-)-=∑LFCgk<0;LFCgk′<0;k≠k′(wgk-wgk′-LFCgkLFCgk′|log10pgk+log10pgk′|)∑wgk-k,

where $w_{\text{gk}}^{+}$ and $w_{\text{gk}}^{-}$ are binary weights of 0 or 1 assigned to the *k*th study for *g*th gene, indicating whether a study is selected for inclusion in aggregate statistics or not, +/− indicate upregulation or downregulation part, ${\vec{w}}_{g}^{+}=\left(w_{\mathrm{g1}}^{+},\ldots ,w_{\text{gK}}^{+}\right)$ and ${\vec{w}}_{g}^{-}=\left(w_{\mathrm{g1}}^{-},\ldots ,w_{\text{gK}}^{-}\right)$. *LFC*_gk_ is the log_2_ fold change and *p*_gk_ the corresponding *p*-value for gene *g* in study *k* obtained from single study DE analysis.

For gth gene, $T_{g\left({\vec{w}}_{g}^{+}\right)}^{+}$ aggregates the information of single study summary statistics (including both *p*-value and effect size) over up-regulated studies (i.e., those studies with *LFC*_gk_ > 0), while $T_{g\left({\vec{w}}_{g}^{-}\right)}^{-}$ aggregates that over down-regulated studies (i.e., those studies with *LFC*_gk_ < 0). The binary weights are used to indicate what studies to include to the aggregate statistics and the optimal weights that maximize the statistics will be searched for each gene. In the proposed aggregate statistics, we simultaneously account for concordant patterns (where*LFC*_gk_ and *LFC*_gk’_ have the same sign), biological significance (estimated as the product of *LFC*_gk_) and statistical significance [estimated as the sum of log_10_(*p*_gk_)]. This will encourage combining studies with the same directionality to find the best evidence for DE, which is consistent with the purpose of meta-analysis to identify more reproducible genes in multiple studies. Similar statistics have been proposed for concordant and discordant analysis of orthologous genes between a pair of species (Domaszewska et al., 2017). From the formula, we can see that the proposed statistic is essentially a weighted average of all study pairs with effect sizes in the same direction. A weighted average of all studies instead of study pairs is an alternative approach but it tends to exclude studies with moderate effect sizes or *p*-values (see a toy example in Supplementary Table 1).

By default, we assume $w_{\text{gk}}^{+}=0$ for studies with *LFC*_gk_ < 0 and $w_{\text{gk}}^{-}=0\text{for}LFC_{\text{gk}}>0$ to avoid conflict between the two statistics. When no studies are up-regulated or down-regulated for a particular gene, we suppress the corresponding $T_{g\left({\vec{w}}_{g}^{+}\right)}^{+}$ or $T_{g\left({\vec{w}}_{g}^{-}\right)}^{-}$ to zero and assign zero weights. The statistics aggregates over study pairs so we need to choose at least two studies to make it meaningful. When only one study is up-regulated or down-regulated, we also suppress the corresponding $T_{g\left({\vec{w}}_{g}^{+}\right)}^{+}$ or $T_{g\left({\vec{w}}_{g}^{-}\right)}^{-}$ to zero.

We then search for the optimal weights to identify the subset of studies that maximize each of the two aggregate statistics. Such optimal weights describe the DE patterns of each gene across studies and provide natural categorization of all genes with potential biological interpretation. The corresponding maximum statistics are defined as:

$$R_{g}^{+}=\max_{{\vec{w}}_{g}^{+}\in W}T_{g\left({\vec{w}}_{g}^{+}\right)}^{+};R_{g}^{-}=\max_{{\vec{w}}_{g}^{-}\in W}T_{g\left({\vec{w}}_{g}^{-}\right)}^{-},$$

where *W* is the pre-defined searching space of weights with aforementioned restrictions. The resulting optimal weights are denoted as ${\vec{w}}_{g}^{+*}$ and ${\vec{w}}_{g}^{-*}$. The biomarkers are then categorized according to the distribution of optimal weights among studies by merging the information of $w_{g}^{+*}$ and $w_{g}^{-*}$, i.e., the final weights ${\vec{w}}_{g}^{*}=\vec{1}\circ {\vec{w}}_{g}^{+*}+\vec{-1}\circ {\vec{w}}_{g}^{-*}$ For example, concordantly up-regulated genes with ${\vec{w}}_{g}^{+*}=\left(0,0,1,1,1\right)$ and ${\vec{w}}_{g}^{-*}$ = (0,0,0,0,0) will be in one category [${\vec{w}}_{g}^{*}=\left(0,0,1,1,1\right)$], while concordantly down-regulated genes with ${\vec{w}}_{g}^{+*}=\left(0,0,0,0,0\right)$ and ${\vec{w}}_{g}^{-*}$ = (0,0,1,1,1) will be in the other category [${\vec{w}}_{g}^{*}=\left(0,0,-1,-1,-1\right)$]. Note that the proposed statistics can describe both up-regulated and down-regulated patterns in the same gene, thus also allowing the detection of discordant genes. In cases both patterns exist and we want to find a dominant pattern in the discordant gene, we can further define *R_g_* = max ($R_{g}^{+},R_{g}^{-}$) and use the corresponding ${\vec{w}}_{g}^{+*}$ or ${\vec{w}}_{g}^{-*}$ for biomarker categorization.

To assess the uncertainty of $R_{g}^{+}$ and $R_{g}^{-}$ and determine DE in meta-analysis, we develop a permutation-based test to calculate the *p*-value and FDR adjusted *p*-value (also known as *q*-value) of the statistics. We permute group labels (i.e., case or control group) in each study *B* times and calculate the maximum statistics in each permuted dataset. For each gene, we obtain two *p*-values corresponding to $R_{g}^{+}$ and $R_{g}^{-}$, respectively:

$$p_{g_{\left(R_{g}^{+}\right)}}^{+}=\frac{\sum_{b=1}^{B}\sum_{g^{\prime }=1}^{G}I\left\{R_{g^{\prime }}^{+\left(b\right)}\geq R_{g}^{+}\right\}+1}{B*G+1};$$

$$p_{g_{\left(R_{g}^{-}\right)}}^{-}=\frac{\sum_{b=1}^{B}\sum_{g^{\prime }=1}^{G}I\left\{R_{g^{\prime }}^{-\left(b\right)}\geq R_{g}^{-}\right\}+1}{B*G+1},$$

where $R_{g^{\prime }}^{+\left(b\right)}$ and $R_{g^{\prime }}^{-\left(b\right)}$ are the maximum statistics for *g*th gene in *b*th (1 ≤ *b* ≤ *B*) permutation. The value of one is added to both numerator and denominator to avoid zero *p*-values. After *p*-values are generated, we further estimate the proportion of null genes π_0_ as:

$${\hat{\pi }}_{0}^{+}=\frac{\sum_{g=1}^{G}I\left\{p_{g_{\left(R_{g}^{+}\right)}}^{+}\epsilon A\right\}}{G*\ell \left(A\right)};{\hat{\pi }}_{0}^{-}=\frac{\sum_{g=1}^{G}I\left\{p_{g_{\left(R_{g}^{-}\right)}}^{-}\epsilon A\right\}}{G*\ell \left(A\right)},$$

normally we choose *A* = [0.5,1] and ℓ(*A*) = 0.5 to estimate the null proportion, following the guidance in the previous methods and the literature of FDR (Storey, 2002; Storey and Tibshirani, 2003; Li and Tseng, 2011). In most cases, the density of *p*-values beyond 0.5 is fairly flat, implying most null *p*-values are located in this region. In practice, depending on the problem, other common choices of A = [0.05,1] or A = [0.025,1] can also be applied. The optimal *A* can be empirically determined by minimizing some loss function, we do not discuss further here and refer readers to Storey (2002), Storey and Tibshirani (2003) for more details.

Then, *q*-values can be calculated as

$$q_{g_{\left(R_{\text{g}}^{+}\right)}}^{+}=\frac{{\hat{\pi }}_{0}^{+}\sum_{b=1}^{B}\sum_{g^{\prime }=1}^{G}I\left\{R_{g^{\prime }}^{+\left(b\right)}\geq R_{\text{g}}^{+}\right\}+1}{B*\sum_{g^{\prime }=1}^{G}I\left\{R_{g^{\prime }}^{+}\geq R_{\text{g}}^{+}\right\}+1},$$

$$q_{g_{\left(R_{\text{g}}^{-}\right)}}^{-}=\frac{{\hat{\pi }}_{0}^{-}\sum_{b=1}^{B}\sum_{g^{\prime }=1}^{G}I\left\{R_{g^{\prime }}^{-\left(b\right)}\geq R_{\text{g}}^{-}\right\}+1}{B*\sum_{g^{\prime }=1}^{G}I\left\{R_{g^{\prime }}^{-}\geq R_{\text{g}}^{-}\right\}+1}$$

Likewise, *p*-value and *q*-value of the dominant pattern statistics *R*_g_ (i.e., *p*_g(R_g_)_ and *q*_g(R_g_)_) can be obtained in the same way. In real data application, we determine DE in meta-analysis using the permuted *p*-value or *q*-value for the dominant pattern. Note that *p*-values and *q*-values of a zero $R_{\text{g}}^{+}$ or $R_{\text{g}}^{-}$ are equal to one.

### Downstream Analysis on Each Identified Categories of Biomarkers

Each transcriptomic study was carefully assessed for inclusion to meta-analysis using objective criteria or systematic quality control methods (Kang et al., 2012). When only expression of mRNA data is available for the K selected transcriptomic studies, we applied our meta-analysis and identified multiple categories of mRNAs at certain BCMC *p*-value or *q*-value cutoffs, each with a unique DE pattern across the studies. DE analysis is useful to narrow down targets but focusing on single gene change across datasets is not sufficient. We still need to conduct further investigation on whether mRNAs belonging to the same category contain unifying biological theme. For each unique category of mRNAs, we then performed pathway enrichment analysis to gain more insights into their unique functions (section “Pathway Enrichment Analysis of mRNA Expression”). When expression data of mRNA, miRNA and lncRNA are all available, we applied our meta-analysis method to each type of transcripts separately and then analyzed each unique category of differentially expressed mRNA, miRNA, and lncRNA (those with the same weight or same cross-study DE pattern) together. Specifically, we performed miRNAs/lncRNAs target gene enrichment analysis (section “miRNAs/lncRNAs Target Gene Enrichment Analysis”) and LncRNA-mRNA and miRNA-mRNA causal regulatory network analysis (section “LncRNA-mRNA and miRNA-mRNA Causal Regulatory Network Analysis”).

#### Pathway Enrichment Analysis of mRNA Expression

For each category of mRNAs with unique DE pattern across the studies, we looked for biological pathways that are enriched in each category of genes more than would be expected by chance. The enriched pathways for each category can infer the unique biological functions only associated with specific study subsets and help generate new hypotheses. The *p*-value for the enrichment of a pathway was calculated using Fisher’s exact test (Upton, 1992) and multiple testing was corrected by Benjamini-Hochberg (BH) procedure (Benjamini and Hochberg, 1995). Multiple popular pathway databases were used including Gene Ontology (GO) (Ashburner et al., 2000), Kyoto Encyclopedia of Genes and Genomes (KEGG) (Kanehisa et al., 2017), Oncogenic signaling Pathways (Sanchez-Vega et al., 2018) and Reactome (Fabregat et al., 2016). Pathways in each pathway database was carefully selected for their relatedness to the problem of interest and small pathways (e.g., pathway size <10) were filtered out for the lack of power. For pathways with topological information available (e.g., pathways in KEGG), we apply the R package *“Pathview”* (Luo and Brouwer, 2013), to display the study-specific information (e.g., weights, effect sizes, etc.) on relevant pathway topology graphs.

#### miRNAs/lncRNAs Target Gene Enrichment Analysis

Going beyond the traditional central dogma, non-coding RNAs such as micro-RNA (or miRNA) and long non-coding RNAs (lncRNA) play important regulatory roles in mRNAs expression (Bartel, 2004; Hubé and Francastel, 2018). To understand whether miRNA/lncRNA target at mRNAs in the same category with unique cross-study DE pattern, we analyzed each unique category of mRNA, miRNA and lncRNA of the same cross-study DE pattern together and performed miRNA/lncRNAs target gene enrichment analysis on each category. Specifically, for each unique category, we first used the miRTarBase database (Chou et al., 2018) and LncRNA2Target v2.0 database (Cheng et al., 2019) to obtain common target genes of each miRNA and lncRNA in this category. We then looked for miRNA/lncRNA with target genes enriched in the gene list falling in the same category more than would be expected by chance. The *p*-value for the enrichment of miRNA/lncRNA was calculated using Fisher’s exact test (Upton, 1992) and multiple testing was corrected by BH procedure (Benjamini and Hochberg, 1995).

#### LncRNA-mRNA and miRNA-mRNA Causal Regulatory Network Analysis

In addition to target gene enrichment analysis, we are also interested in investigating the causal regulatory relationship among the various types of transcripts in the same category using network analysis. For each unique category of mRNA and lncRNA with the same cross-study DE pattern, we followed the MSLCRN pipeline to perform module-specific lncRNA-mRNA regulatory network analysis (Zhang et al., 2019). The MSLCRN pipeline starts by using WGCNA (Langfelder and Horvath, 2008) to construct lncRNA-mRNA co-expression networks and identify modules that contain both lncRNA and mRNA. For each lncRNA-mRNA module, parallel IDA (Le et al., 2016) is then applied to learn the causal structure and estimate the causal effect of lncRNA on mRNA. IDA consists of two main steps. It first uses a parallel version of the PC algorithm (Spirtes et al., 2000; Kalisch and Bühlman, 2007; Le et al., 2016), commonly used approach for learning the causal structure of a Bayesian network, to obtain the directed acyclic graphs (DAGs) for each module. Then, the causal effect of lncRNAs on mRNAs (i.e., the lncRNA ≥ mRNA directed edges in the DAG) are estimated by applying do-calculus (Pearl, 2000), causal calculus that uses Bayesian conditioning to generate probabilistic formulas for the causal effect. Lastly, the module-specific causal regulatory networks are integrated to form the global lncRNA-mRNA causal regulatory network and visualized using Cytoscape (Shannon et al., 2003). In constructing the regulatory network, we use absolute values of the causal effects cutoffs to assess the regulatory strengths and confirm the regulatory relationships. More details on the use of MSLCRN to infer causal regulatory network can be found in Zhang et al. (2019). Module-specific miRNA-mRNA causal regulatory networks can be obtained in a similar way using the same tool.

## Simulation

We conduct simulation studies to evaluate the performance of our method in biomarker detection and categorization when compared to AW-Fisher (Li and Tseng, 2011), FEM and REM methods (Choi et al., 2003). Only power is assessed for FEM and REM methods since they do not categorize biomarkers. We assume a total of *G* = 2000 genes expressed in *K* = 5 studies, each study has a total sample size of *n* = 100, evenly split into control and case groups $\left(n_{\text{case}}=n_{\text{control}}=\frac{n}{2}=50\right)$. The details on how data are simulated are described below:

1. We generate 800 genes with 40 gene clusters (20 genes in each cluster) and another 1,200 genes that do not belong to any cluster. The cluster indexes for each gene g (1 ≤ *g* ≤ 2000) is randomly sampled.
2. For genes in cluster *c* (1 ≤ *c* ≤ 40) and study *k*(1 ≤ *k* ≤ 5), we first generate a covariance matrix according to inverse Wishart distribution $\Sigma_{ck}^{\prime }∼W^{-1}\left(\Psi ,60\right)$, where Ψ = 0.5*I*_20×20_ + 0.5*J*_20×20_, *I* is the identity matrix and *J* is the matrix with all elements equal to one. Then, we standardized $\Sigma_{ck}^{\prime }$ into *Σ*_*ck*_ to make sure all the diagonal elements are one.
3. We sample baseline gene expression levels of the 20 genes in cluster *c* for sample *i* in study k by ${\left(X_{g_{\mathrm{c1}}\mathrm{ik}}^{\prime },\ldots ,X_{g_{\mathrm{c20}}\mathrm{ik}}^{\prime }\right)}^{T}∼MVN\left(0,\Sigma_{ck}\right)$, where 1 ≤ *i* ≤ *n* and 1 ≤ *k* ≤ *K*. For those 1200 genes that are not in any cluster, we sample the baseline gene expression level independently from $N\left(0,\sigma_{\text{k}}^{2}\right)$, where 1 ≤ *k* ≤ 5 and σ_k_∼*Unif*(σ−0.2,σ + 0.2) with σ = 2.
4. Denote by δ_gk_ ∈ {0,1,−1} that gene *g* is non-DE, up-regulated or down-regulated in study *k*. We assume the first 800 genes to be DE genes divided into four mutually exclusive parts:
  (1) Concordantly up-regulated genes (*N* = 225): randomly sample δ_gk_ ∈ {0,1,−1} such that $\sum_{\text{k}}I_{\left\{\delta_{\text{gk}}=1\right\}}\geq 2$ and $\sum_{\text{k}}I_{\left\{\delta_{\text{gk}}=-1\right\}}\leq 1$.
  (2) Concordantly down-regulated genes (*N* = 225): randomly sample δ_gk_ ∈ {0,1,−1} such that $\sum_{\text{k}}I_{\left\{\delta_{\text{gk}}=-1\right\}}\geq 2$ and $\sum_{\text{k}}I_{\left\{\delta_{\text{gk}}=1\right\}}\leq 1$.
  (3) Discordant genes with both up-regulated and down-regulated patterns (*N* = 150): randomly sample δ_gk_ ∈ {0,1,−1} such that $\sum_{\text{k}}I_{\left\{\delta_{\text{gk}}=1\right\}}\geq 2$ and $\sum_{\text{k}}I_{\left\{\delta_{\text{gk}}=-1\right\}}\geq 2$.
  (4) Other genes that are DE in only one study without any concordant patterns (*N* = 200): we randomly sample δ_gk_ ∈ {0,1,−1} such that $\sum_{\text{k}}\left|\delta_{\text{gk}}\right|=1$.
5. To simulate effect size for DE genes in each study (when δ_gk_ ≠ 0), we sample from a uniform distribution μ_gk_∼*Unif*(1,3). The gene expression level *X*_gik_ are assumed to be $X_{\text{gik}}^{\prime }$ for control samples and $X_{\text{gik}}=X_{g\left(i+n/2\right)k}^{\prime }+\mu_{\text{gk}}\cdot \delta_{\text{gk}}$ for case samples, where 1 ≤ *g* ≤ 2000, 1 ≤ *i* ≤ *n*/2, and 1 ≤ *k* ≤ 5.

To assess power and biomarker categorization performance, we focus on DE genes in the first three categories of genes with concordant patterns in at least two studies (*N* = 600). We also simulate additional scenario with smaller sample size and variance: *n* = 20 & σ = 1, results are included in the Supplement (Supplementary Figure 1 and Supplementary Table 2).

**FIGURE 2:**
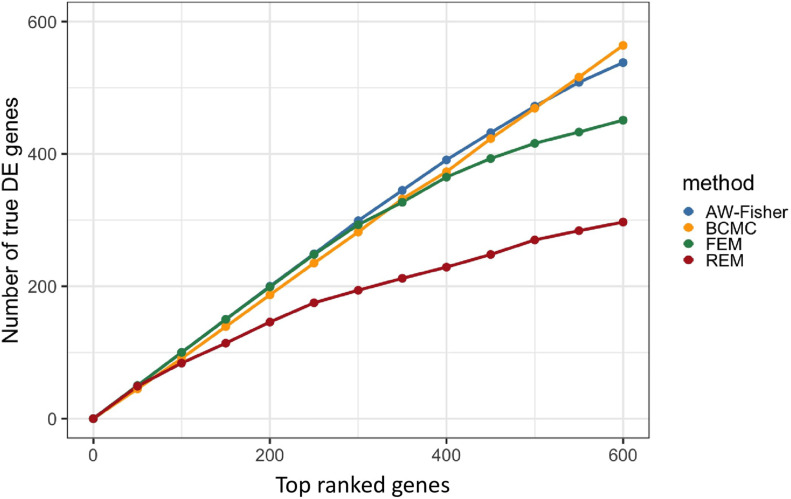
Plot of the number of true DE genes vs. top ranked genes by *p*-value of each method.

Figure 2 shows the number of true DE genes detected among the top genes ranked by *p*-value for each method. BCMC is more powerful than AW-Fisher and FEM/REM by detecting more true DE genes among the top ranked genes. Table 1 summarizes the number of true DE genes detected as well as with correct weight pattern in each of the three categories of DE genes identified by each method. BCMC and FEM detect more true DE genes than AW-Fisher for concordant genes. Due to the model restriction, FEM and REM fail to detect most discordant genes. AW-Fisher is equally powerful as BCMC in detecting discordant genes, however, it ignores the directionality of effects, and thus assigns the incorrect weights to genes with both up-regulated and down-regulated patterns (basically they fail to distinguish *w* = −1 from *w* = 1). Our method detects these discordant DE genes while at the same time assigns the correct weights categorizing these genes.

**TABLE 1:** Summary of number of true DE genes detected and with correct weight patterns by the four methods in each of the three categories of DE genes described in the simulation setting.

Methods	BCMC		AW-Fisher		FEM	REM
DE Gene categories	Number of true DE genes	Number of true DE genes with correct weight	Number of true DE genes	Number of true DE genes with correct weight		
Concordant up (*N* = 225)	206	116	195	106	203	151
Concordant down (*N* = 225)	210	119	195	108	201	144
Discordant (*N* = 150)	148	135	148	0	47	2
Total (*N* = 600)	564	370	538	214	451	297

## Real Data Application

### Gene Expression Analysis in Pan-Gynecologic (Pan-Gyn) Studies

We applied our method to the gene expression data of TCGA Pan-Gyn studies including high-grade serous ovarian cystadenocarcinoma (OV), uterine corpus endometrial carcinoma (UCEC), cervical squamous cell carcinoma and endocervical adenocarcinoma (CESC), uterine carcinosarcoma (UCS), and invasive breast carcinoma (BRCA) (Berger et al., 2018). Berger et al. (2018) identified 23 genes (e.g., BRCA1, PTEN, TP53, etc.) that were mutated at higher frequency across all Pan-Gyn cancers than non-Gyn cancers, highlighting the similarities across Pan-Gyn cohort. We focused on 19 of these genes and split samples in each study into a mutation “carrier” group and a mutation “non-carrier” group depending on whether subjects gained mutations in at least one of the genes (Supplementary Figure 2). Since no or very few samples were assigned to the mutation carrier group for UCS (*N*_*mutation*_ = 0) and UCEC (*N*_*mutation*_ = 8), we excluded those two studies and restricted our meta-analysis to only three gynecologic cancer types (i.e., number of studies *K* = 3) including OV (mutation carrier vs. non-carrier: 217/90), BRCA (692/408) and CESC (109/197). The purpose is to detect differentially expressed genes between mutation carrier and non-carrier groups and categorize them according to their cross-study DE patterns. We found the overall survival differed significantly between the two groups for each cancer type (Supplementary Figures 3–5). This implied the differentially expressed biomarkers between these two groups can have potential prognostic values related to mutational processes and serve as optimal therapeutic intervention targets (Helleday et al., 2014; Lawrence et al., 2014).

The RNA-seq data in Transcripts Per Million (TPM) values of each cancer type were downloaded from LinkedOmics (Vasaikar et al., 2018). We first merged the three datasets by matching the gene symbols and removed genes with mean TPM < 5. A total of 9,900 mRNAs remained and were log_2_ transformed for analysis. We performed DE analysis by limma (Ritchie et al., 2015) and obtained the *p*-value and LFC from each of the three studies. We then performed meta-analysis using BCMC and the other methods.

**FIGURE 3:**
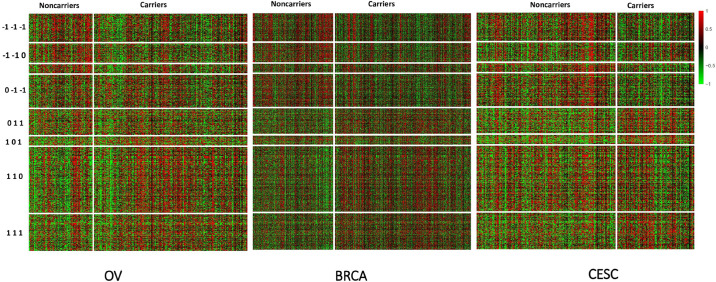
Heatmap of standardized expression values of differentially expressed genes (BCMC *q* < 0.05) sorted by weight patterns for the Pan-Gyn cancer example.

**FIGURE 4:**
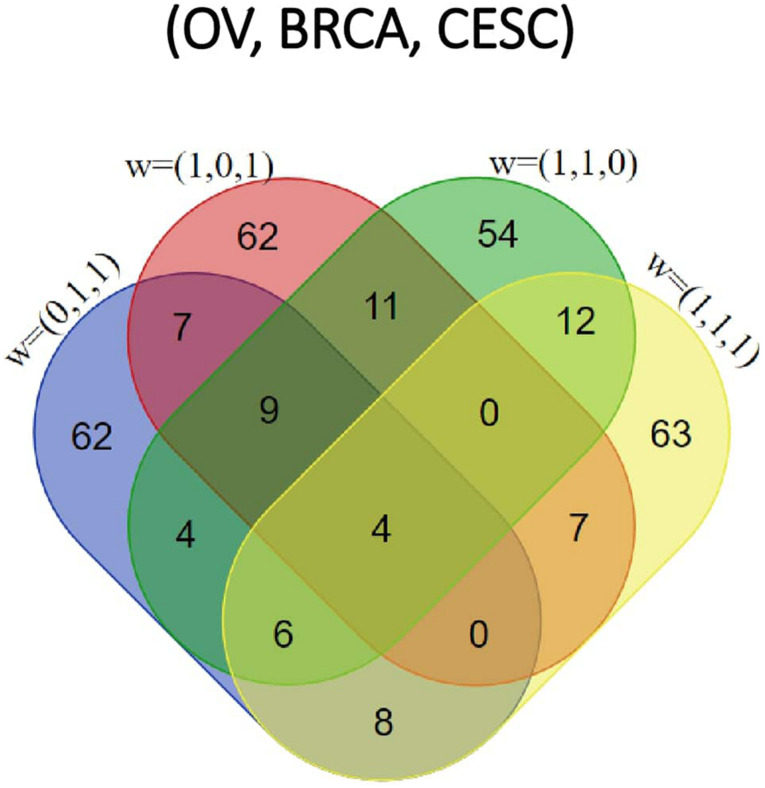
Venn diagram of top 100 pathways enriched by each of the four categories [$\left|w_{\text{g}}^{*}\right|=\left(0,1,1\right),\left(1,0,1\right),\left(1,1,0\right),\text{and}\left(1,1,1\right);$ corresponding to OV, BRCA and CESC, respectively] for the Pan-Gyn study example.

**FIGURE 5:**
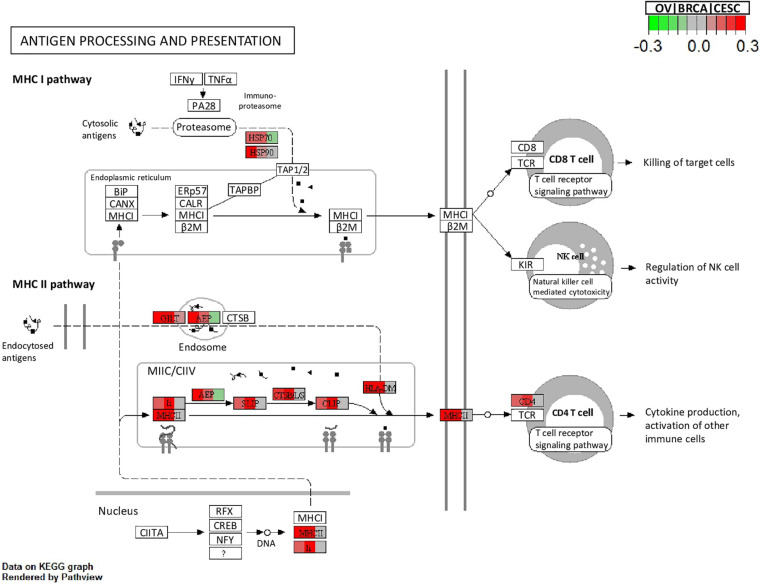
Visualization of the topology plot of a KEGG pathway “Antigen processing and presentation” enriched by the genes with $\left|w_{\text{g}}^{*}\right|=\left(1,1,0\right)$ (corresponding to OV, BRCA and CESC, respectively) for the Pan-Gyn example. Each box that represents a gene is split into three parts to represent the three studies. Colors indicate the signed LFC of the mapped DE genes in the three studies.

All methods detected thousands of DE genes at both *q*-value cutoffs (for BCMC, *q*-value for dominant pattern was used so we focused on concordant genes only), which is common in Pan-cancer studies (Table 2). It becomes imperative task to partition these DE genes into smaller subsets by cross-study DE patterns before performing downstream analysis. BCMC categorized these DE biomarkers (*q* < 0.05) into eight groups according to the optimal weight assignments, each displaying a unique expression pattern across the different studies (Figure 3 and Supplementary Table 3). We then merged genes with equal |${\vec{w}}_{\text{g}}^{*}$| into the same group (i.e., genes with ${\vec{w}}_{\text{g}}^{*}=\left(0,1,1\right)$ and those with ${\vec{w}}_{\text{g}}^{*}=\left(0,-1,-1\right)$ are merged into the same group, allowing both up-regulated and down-regulated genes in the same pathway) and performed pathway enrichment analysis on each of the four merged groups using four pathway databases: GO (Ashburner et al., 2000), KEGG (Kanehisa et al., 2017), Oncogenic (Sanchez-Vega et al., 2018) and Reactome (Fabregat et al., 2016). The top 100 pathways enriched by each category have little overlap partly validating our speculation in motivation that the different categories of biomarkers may play different functional roles (Figure 4). For example, top pathways for $\left|{\vec{w}}_{\text{g}}^{*}\right|=\left(1,0,1\right)$ (i.e., DE in OV and CESC but not in BRCA) are mainly involved in cell junction and adhesion related functions (Supplementary Table 4 in Supplemental File 1). Top pathways for $\left|{\vec{w}}_{\text{g}}^{*}\right|=\left(1,1,0\right)$ (i.e., DE in OV and BRCA but not in CESC) are mainly involved in immune and defense response. Figure 5 shows the topology of one example KEGG pathway “Antigen processing and presentation” enriched by the genes with $\left|{\vec{w}}_{\text{g}}^{*}\right|=\left(1,1,0\right)$. The highlighted DE genes showed strong DE signals (signed LFC) in OV and BRAC but not in CESC. These genes colocalized and interacted with each other as a functional unit inside the pathway.

**TABLE 2:** Summary of numbers of DE genes detected by each method at different cutoffs for the Pan-Gyn study example. For BCMC, *q*-values for the dominant pattern are used.

Methods				
*q*-value	BCMC	AW-Fisher	FEM	REM
*q* < 0.05	1,345	3,113	2,866	983
*q* < 0.15	3,931	4,743	4,342	1,641

These unique gene sets of different cross-cancer DE patterns and the associated pathways enriched help gain more insights into the homogeneous and heterogenous molecular mechanism of different Gynecologic cancer and assist the development of useful diagnostic and therapeutic strategies common or specific to cancer types. Understanding commonality and difference in drug targets can also guide the drug repurposing strategy in cancer drug development (Li et al., 2021).

### Integrative Analysis of mRNA, lncRNA, and miRNA in Pan-Kidney Studies

We also used BCMC to perform integrative analysis of three different types of transcripts (mRNA, lncRNA, and miRNA) in the TCGA Pan-Kidney cohort including kidney chromophobe (KICH), kidney renal clear cell carcinoma (KIRC), and kidney renal papillary cell carcinoma (KIRP). LncRNA and miRNA have been found playing important regulatory roles on gene expression in kidney cancers (Linehan et al., 2010; Linehan, 2012; Ricketts et al., 2018). The integrative analysis of these multi-omics data provides additional insights into the biological mechanism underlying the multiple histologic subtypes of kidney cancers. We aimed to detect the differentially expressed biomarkers (mRNA, miRNA, or lncRNA) that drive the progression of kidney cancer by comparing samples from early pathologic stage (stage I and II) to late stage (stage III and stage IV) for three kidney cancer types (i.e., number of studies *K* = 3) and investigating the regulatory relationships among these biomarkers. Number of subjects in the two pathologic stages of each kidney cancer available in mRNA, miRNA and lncRNA expression data were summarized in Supplementary Table 5.

We downloaded mRNA (in Reads Per Kilobase of transcript per Million mapped reads or RPKM) and miRNA (in Reads Per Million mapped reads or RPM) sequencing data from LinkedOmics (Vasaikar et al., 2018) and lncRNA sequencing data (in RPM) from The Atlas of Noncoding RNAs in Cancer (TANRIC) (Li et al., 2015) for all the three kidney cancer subtypes. We first merged the three subtypes by matching RNA symbols/IDs. We then separately filtered each of the three types of biomarkers by removing mRNAs with mean RPKM < 5, lncRNAs with mean RPM < 0.1, and miRNAs with mean RPM = 0, followed by log_2_ transformation. A total of 15,332 mRNAs, 2,415 lncRNAs and 719 miRNAs remained for analysis. We performed DE analysis by limma (Ritchie et al., 2015) in each study and then meta-analysis to categorize biomarkers according to cross-study DE patterns for each RNA species. For different types of RNA belonging to the same category, we further performed miRNA target gene enrichment analysis and lncRNA-mRNA causal regulatory network analysis to understand their complex interacting relationships in kidney cancer.

**FIGURE 6:**
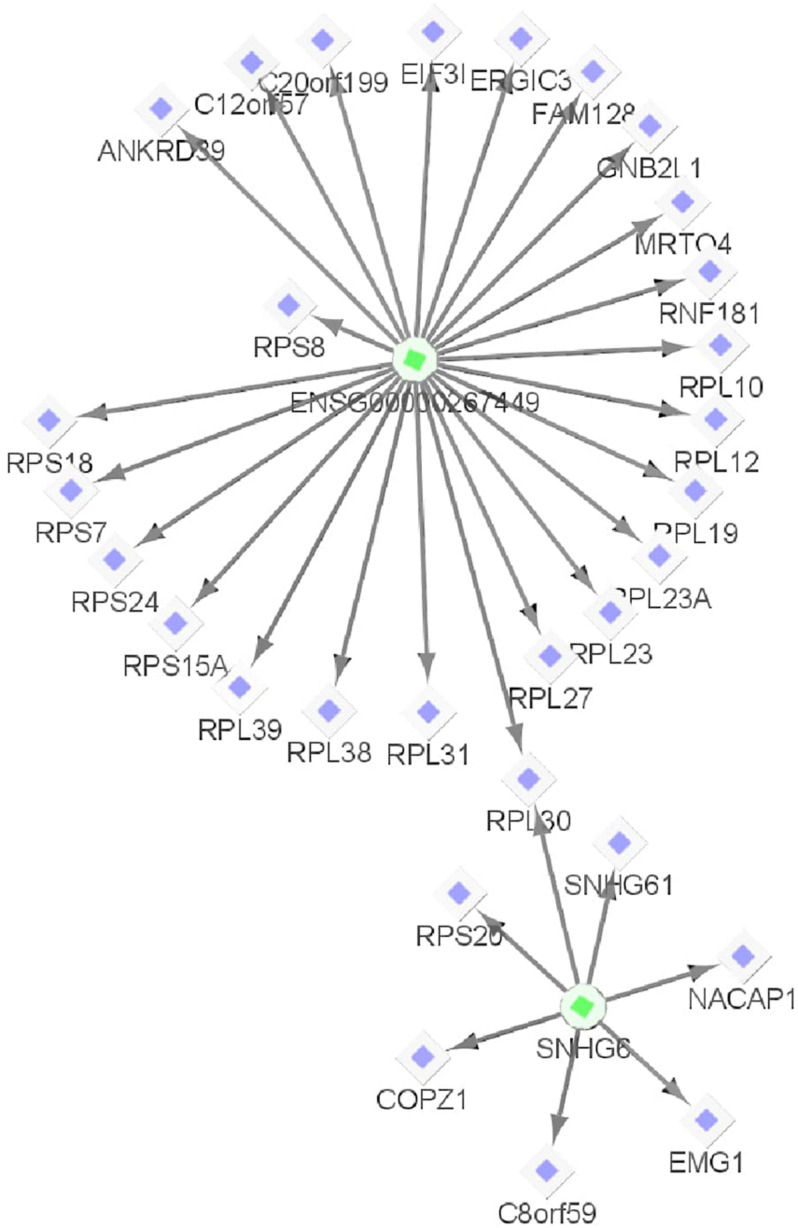
One example lncRNA-mRNA regulatory network identified from biomarkers with | $w_{\text{g}}^{*}|=\left(1,1,1\right)$ (corresponding to KICH, KIRC, and KIRP, respectively) for the Pan-Kidney example. The circle shapes represent lncRNAs highlighted in green and diamond shapes represent mRNAs highlighted in purple. The arrows indicate the network relationships between lncRNAs and mRNAs.

Both BCMC and AW-Fisher methods detected thousands of differentially expressed biomarkers (including mRNA, lncRNA, and miRNA) at both *q*-value cutoffs with high proportion of overlap (Table 3). Biomarkers detected by BCMC tend to have both significant *p*-values and large effect sizes in the studies indicated by optimal weights (Supplementary Figure 6). These biomarkers (*q* < 0.05) were partitioned into eight categories by different weight patterns (Supplementary Table 6). We merged biomarkers with the same $\left|{\vec{w}}_{g}^{*}\right|$ into the same group. We focused on the group with $\left|{\vec{w}}_{\text{g}}^{*}\right|=\left(1,1,1\right)$ to understand the common multi-omics regulatory among all histologic subtypes of kidney cancer and performed downstream analysis. In miRNA target gene enrichment analysis, we found the target gene sets of two DE miRNAs “miR-655” and “miR-326” were enriched in the DE gene list in the same group (*p* < 0.05; Supplementary Table 7 in the Supplementary File 1), implying the potential regulatory relationship between different biomarker types consistent in all kidney cancer subtypes. The gene *ATAD2* targeted by miR-655 was reported as a prognostic marker for kidney disease (Chen et al., 2017). In causal network analysis, we identified two lncRNA-mRNA regulatory networks (Supplementary Figure 8 and Supplementary Table 8). Figure 6 shows the network with two hub lnRNAs, the hub lncRNA ENSG00000267449 and several mRNAs belonging to the ribosomal protein family in the same network were found consistently differentially expressed in all three subtypes, implying their potentially joint role in promoting the development of kidney cancers (Zhou et al., 2015; Dolezal et al., 2018).

**TABLE 3:** Summary of number of differentially expressed biomarkers among each of the three RNA species detected by each method at different cutoffs for the Pan-Kidney study example. For BCMC, *q*-values for the dominant pattern are used.

Type of biomarkers	mRNA		lncRNA		miRNA	
*q*-value	BCMC	AW-Fisher	BCMC	AW-Fisher	BCMC	AW-Fisher
*q* < 0.05	7,317	9,472	764	1,281	239	283
Intersection	6,391		622		206	
*q* < 0.15	11,810	11,440	1,468	1,464	358	358
Intersection	10,057		1,244		292	

These results demonstrate the power of our method to detect biomarkers of different types in Pan-cancer meta-analysis and to categorize them into functionally relevant biomarkers by DE patterns, which could suggest commonalities and differences in underlying mechanisms of multiple cancer types.

## Discussion

In this paper, we proposed a novel meta-analysis method for candidate biomarker detection in multiple transcriptomic studies that further categorizes biomarkers by concordant patterns as well as by biological and statistical significance across studies. Numerous downstream analysis tools including pathway analysis and causal network analysis are applied to each category of biomarkers with either single or multiple types of RNA species. Simulations and real data application to two Pan-cancer multi-omics studies showed the advantage of our method in classifying differentially expressed biomarkers into classes with unique biological functions and relationships that can be further investigated in future studies.

Meta-analysis is a set of statistical analytical methods and tools that combine multiple related studies to improve power and reproducibility over a single study. In recent years, we have witnessed the development of many useful meta-analysis methods applied to genomic studies for different biological purposes (Choi et al., 2003; Shen and Tseng, 2010; Li and Tseng, 2011; Huo et al., 2016, 2020; Kim et al., 2016, 2018; Zhu et al., 2017; Ma et al., 2019; Zeng et al., 2020). Genomic data is usually of high dimension and the between study heterogeneity is large due to both technological and cohort effects. In addition to improving power, post-hoc categorization of biomarkers into smaller subsets by cross-study patterns for subsequent analysis is important in genomic meta-analysis. Our meta-analysis method that aggregates over both *p*-value and effect size is a fast and intuitive solution for this purpose. Compared to other popular meta-analysis methods that include biomarker categorization, our method considers concordant pattern, and biological and statistical significance simultaneously. By calculating statistics separately for up-regulated and down-regulated parts, we can detect both concordant genes that have consistent patterns across all studies and discordant genes that are up/down regulated in some studies while down/up regulated in others. Both of these kinds of genes can be of interest in Pan-cancer analysis. For example, high expression of some genes might worsen the prognosis of all cancer types, while high expression of other genes might worsen prognosis for some cancers but be beneficial to other cancer types.

Our method also applies to the scenario when there is more than one RNA species present and proposes to jointly analyze different types of biomarkers under the same category for more biological insights. As more omics data are accumulated in the public domain, similar strategies can be applied for integrative analysis, for example with epigenomic (e.g., DNA methylation, histone modification), proteomic and metabolomic data. Unique features of each omics data type need to be addressed and will be considered as a future direction to extend our method.

Like most other two-stage meta-analysis methods, our method is based on summary measures such as *p*-values and log_2_ fold changes from each study. In addition, the method assigns a single optimal weight to each gene without quantifying the uncertainty in weight assignment. A more comprehensive Bayesian hierarchical model can be applied to raw data and summary measures to better capture the stochasticity and provide soft weight assignment. Our method requires the DE genes to be concordant in at least two studies to be detected, consistent with the purpose of meta-analysis in prioritizing more reproducible biomarkers. As the number of studies becomes large, the likelihood of being differentially expressed in only one study decreases. Thus, we expect the method to perform well as the number of studies increases. Since the method relies on summary measures, increasing the number of studies will not materially increase the computational burden. Additionally, use of more sophisticated parallel computing techniques will improve the speed of permutation tests. An R package called “BCMC” is available at https://github.com/kehongjie/BCMC to implement our method.

## Data Availability Statement

Publicly available datasets were analyzed in this study. This data can be found here: https://github.com/kehongjie/BCMC.

## Author Contributions

ZY and HK developed the method, performed the analysis, and wrote the manuscript. TM supervised the project and took the lead in editing the manuscript. SC, RC-C, XH, JZ, JD, and DM contributed to manuscript writing and polishing. All authors provided critical feedback and helped shape the research, analysis and manuscript.

## Conflict of Interest

The authors declare that the research was conducted in the absence of any commercial or financial relationships that could be construed as a potential conflict of interest.
