# Supplementary material for: Biomarker Categorization in Transcriptomic Meta-Analysis by Concordant Patterns With Application to Pan-Cancer Studies
Source: Front Genet. 2021 Jul 2;12:651546. doi: 10.3389/fgene.2021.651546 (PMC8283696; doi:10.3389/fgene.2021.651546)
Supplement: Supplementary file 2 [file Table_2.docx]

Supplementary Material

# Supplementary Tables

**Table S1.** A toy example of optimal weights determined by simple weighted average of studies (SWA= $\frac{\sum_{\boldsymbol{k=1}}^{\boldsymbol{K}} \boldsymbol{w}_{\boldsymbol{gk}}|LFC_{gk}\boldsymbol{||}\boldsymbol{log}_{\boldsymbol{10}}\boldsymbol{P}_{\boldsymbol{gk}}\boldsymbol{|}}{\sum_{\boldsymbol{k=1}}^{\boldsymbol{K}} \boldsymbol{w}_{\boldsymbol{gk}}}$) and our proposed method BCMC for different effect sizes and p-values. We use -log10(p) = 5, 3, 1 and LFC=1, 0.5, 0.3 to indicate strong, moderate and weak DE evidence, respectively. SWA tends to exclude studies with moderate effect size or p-value in their optimal weight selection (study 3 in gene 2-4). The optimal weights selected by each method are highlighted in red.

|  | **Study 1** | | **Study 2** | | **Study 3** | | **SWA** | | **BCMC** | |
| --- | --- | --- | --- | --- | --- | --- | --- | --- | --- | --- |
|  | **-log10(p)** | **LFC** | **-log10(p)** | **LFC** | **-log10(p)** | **LFC** | **w= (1,1,1)** | **w= (1,1,0)** | **w= (1,1,1)** | **w= (1,1,0)** |
| Gene1 | 5.00 | 1.00 | 5.00 | 1.00 | 5.00 | 1.00 | 5.00 | **5.00** | **10.00** | 5.00 |
| Gene2 | 5.00 | 1.00 | 5.00 | 1.00 | 3.00 | 1.00 | 4.33 | **5.00** | **8.67** | 5.00 |
| Gene3 | 5.00 | 1.00 | 5.00 | 1.00 | 3.00 | 0.50 | 3.83 | **5.00** | **6.00** | 5.00 |
| Gene4 | 5.00 | 1.00 | 5.00 | 1.00 | 1.00 | 0.30 | 3.43 | **5.00** | 4.53 | **5.00** |

**Table S2.** Summary of number of true DE genes detected and with correct weight patterns by the four methods in each of the three categories of DE genes ((1)-(3)) described in the simulation setting for the scenario with $n=20$ & $\sigma=1$.

| Methods  DE  Gene  categories | BCMC | | AW Fisher | | FEM | REM |
| --- | --- | --- | --- | --- | --- | --- |
|  | Number of true DE genes | Number of DE genes with correct pattern | Number of true DE genes | Number of DE genes with correct pattern |  |  |
| Concordant up (N=225) | 201 | 105 | 193 | 97 | 187 | 140 |
| Concordant down (N=225) | 210 | 110 | 190 | 92 | 197 | 155 |
| Discordant (N=150) | 147 | 120 | 147 | 0 | 37 | 3 |
| Total (N=600) | 558 | 335 | 530 | 189 | 421 | 298 |

**Table S3.** The eight weight categories of DE biomarkers identified by BCMC at q-value <0.05 for the Pan-Gyn example.

| **Weight Category** | **Number of DE genes** |
| --- | --- |
| **(OV, BRCA, CESC)** |  |
| -1-1-1 | 168 |
| -1-10 | 113 |
| -10-1 | 51 |
| 0-1-1 | 195 |
| 011 | 153 |
| 101 | 53 |
| 110 | 394 |
| 111 | 218 |

**Table S4.** Pathway enrichment analysis results for the four weight categories (|$w_{g}^{*}|=(0,1,1$), (1,0,1), (1,1,0), (1,1,1); corresponding to OV, BRCA and CESC, respectively) for the Pan-Gyn example **(**See Supplementary file1).

**Table S5.** Summary of number of participants in two pathological stages for Pan-kidney cohort.

| Numbers of participants in different omics data type | KICH  (early/late) | KIRC  (early/late) | KIRP  (early/late) | Total |
| --- | --- | --- | --- | --- |
| mRNA | 66(46/20) | 531(324/207) | 260(193/67) | 857 |
| micro RNA | 66(46/20) | 514(308/206) | 216(194/67) | 841 |
| long non-coding RNA | 66(46/20) | 447(206/187) | 184(132/52) | 697 |

**Table S6.** The eight weight categories of DE biomarkers identified by BCMC at q-value < 0.05 for each type of RNA species for the Pan-Kidney example.

| **Type of biomarkers** | **mRNA** | **lncRNA** | **miRNA** |
| --- | --- | --- | --- |
| **Weight category**  **(KICH, KIRC, KIRP)** | **Number of DE biomarkers** | | |
| -1-1-1 | 1003 | 129 | 9 |
| -1-10 | 652 | 45 | 12 |
| -10-1 | 708 | 180 | 9 |
| 0-1-1 | 846 | 147 | 12 |
| 011 | 958 | 46 | 25 |
| 101 | 811 | 40 | 52 |
| 110 | 953 | 111 | 35 |
| 111 | 1386 | 66 | 85 |

**Table S7.** miRNA target enrichment analysis results for |$w_{g}^{*}|=(1,1,1)$ **(**corresponding to the study KICH, KIRC and KICH, respectively) for the Pan-Kidney example **(**See Supplementary file1).

**Table S8**. Important lncRNAs and mRNAs in the regulatory networks (Figure S8) constructed based on the biomarkers with |$w_{g}^{*}|$= (1,1,1) for the Pan-Kidney cohort.

| **Network** | **Types of RNA** | **Names** | **Description/Biological functions** | **Reference** |
| --- | --- | --- | --- | --- |
| **Network1** | **lncRNA** | SNHG6 | Small Nucleolar RNA Host Gene 6/The up-regulation of SNHG6 is associated with poor overall survival in renal cell carcinoma. | An et al, 2018 [1] |
|  | **mRNA** | RPS20 | Ribosomal protein S20/Significantly up-regulated in tumor kidney tissues compared to the normal kidney tissues | Wu et al., 2020 [2] |
|  |  | RPL30 | Ribosomal protein L30/Elevation of protein synthesis in cancer proliferation. | Zhou et al., 2015 [3] |
|  | **lncRNA** | ENSG00000267449 | Novel transcript, antisense to BCAS3 | Bryzghalov, Szcześniak, & Makałowska, 2016 [4] |
|  | **mRNA** | GNB2L1 | Guanine nucleotide-bingding protein subunit beta-2-like 1/A high frequency mutation of GNB2L1 targets PI3K/AKT pathway activation leading to increase proliferation, invasion and metastases in RCC. | Guo et al., 2015 [5] |
|  |  | RNF181 | Ring finger protein-181/It has a RING-finger domain and E3 enzyme activity, dysregulating ubiquitin-proteasome pathway to develop kidney disease. | Tan et al., 2015 [6] |
|  |  | RPL31, RPL38, RPL39, RPS24 | Ribosomal protein family/Elevate protein synthesis in cancer proliferation. | Dolezal et al., 2018 [7], Zhou et al., 2015 [3] |
| **Network2** | **lncRNA** | ENSG00000228487 | Novel Transcript, antisense To RALGPS1 | Stelzer et al., 2016 [8] |
|  |  | LINC00957 | An epithelial-mesenchymal transition (EMT) related lncRNA. High expression of LINC00957 is associated with higher overall survival for ccRCC patients. | Zhong et al., 2020 [9] |
|  |  | ENSG00000233255 | Novel transcript, antisense to AC019181.2 | Perron et al., 2017 [10] |
|  |  | ENSG00000260579 | Novel transcript, antisense to TM6SF1 | Stelzer et al., 2016 [8] |
|  |  | ENSG00000254528 | Novel transcript, antisense to FXYD6 | Yates et al., 2020 [11] |
|  |  | ENSG00000261399 | Novel transcript, antisense to MAPK8IP3 | Yates et al., 2020 [11] |
|  | **mRNA** | TRIM2 | Tripartite motif-containing protein 2/The down-regulation of TRIM2 suppresses cell proliferation, migration, and invasion that leads to poor survival in clear cell renal cell carcinoma. | Xiao et al.,2018 [12] |
|  |  | RNF128 | Ring finger protein-128/A members of Goliath family for controlling the development of T-cell clonal anergy that observed in tumor kidney tissue. | Anandasabapathy et al., 2003 [13], Jin et al., 2011[14] |
|  |  | HDGF | Hepatoma-derived growth factor/The expression of HDGF is essential for kidney malformation. | Sun et al., 2018 [15] |
|  |  | PACRG | Parkin coregulated gene protein/The down-regulation of PACRG is associated with shorter disease-free survival and overall survival in the clear cell renal cell carcinomas. | Toma et al., 2013 [16] |

## 2. Supplementary Figures


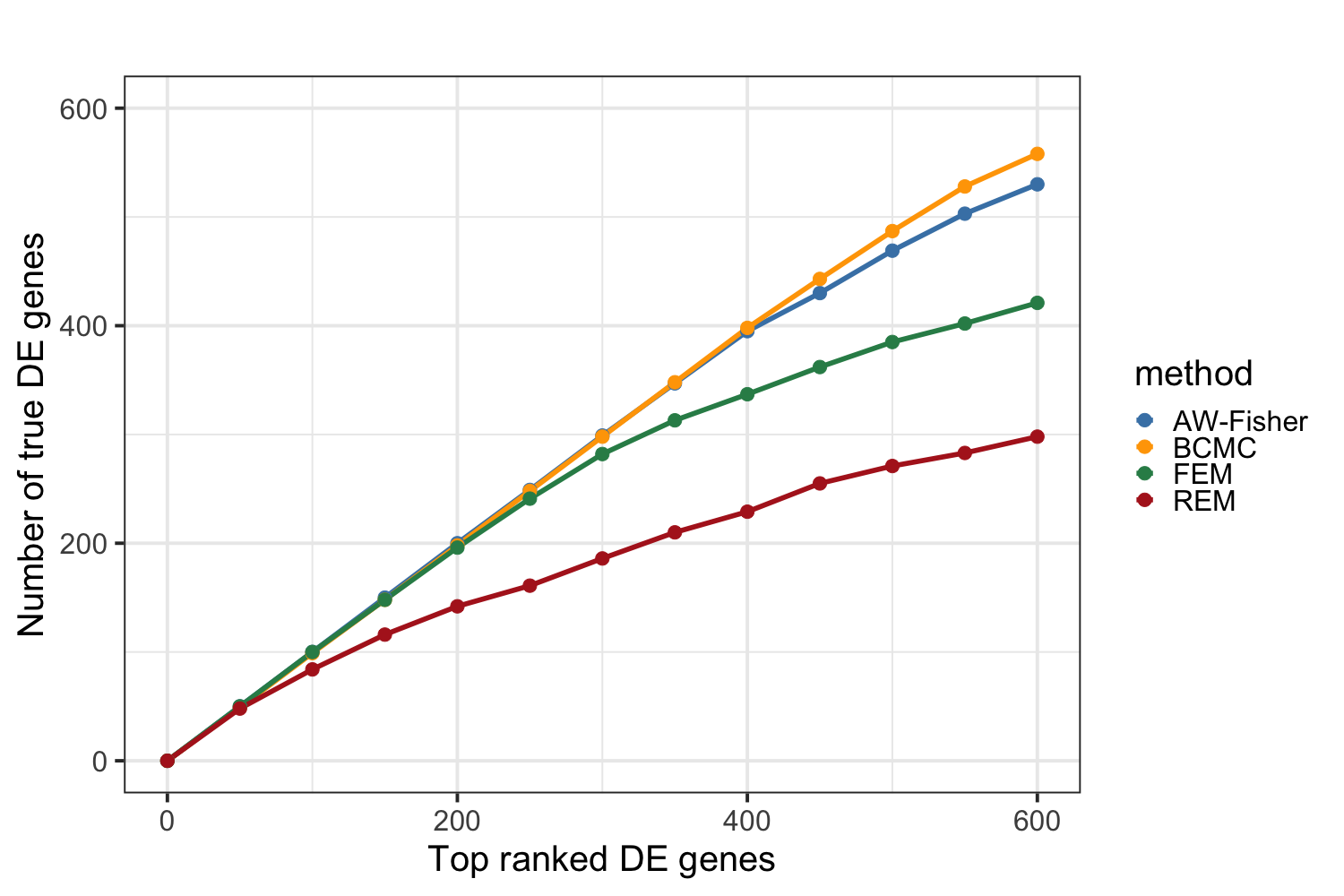


**Figure S1.** Plot of the number of true DE genes in the first three categories vs. top ranked genes by p-value of each method for the scenario with $n=20 \& \sigma=1$.

**Figure S2.** Mutation frequencies of 19 previously reported Pan-Gyn specific mutation signature genes among all samples of Pan-Gyn cohort.

##
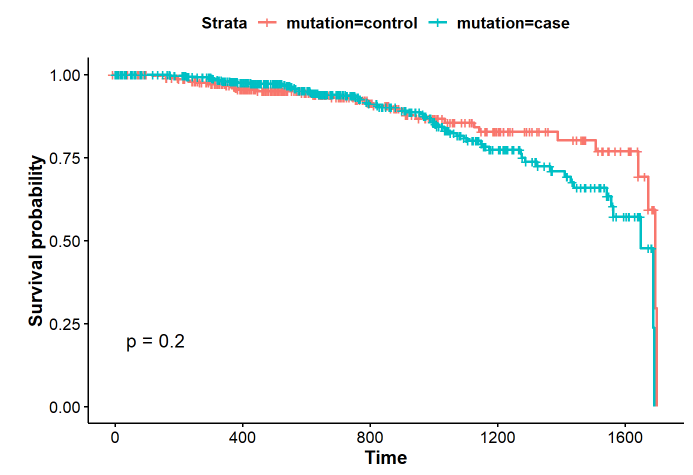


**Figure S3.** Comparison of Kaplan Meier curves of the overall survival between mutation carrier group (case) and mutation non-carrier group (control) in OV. The p-value shown is from the log-rank test.


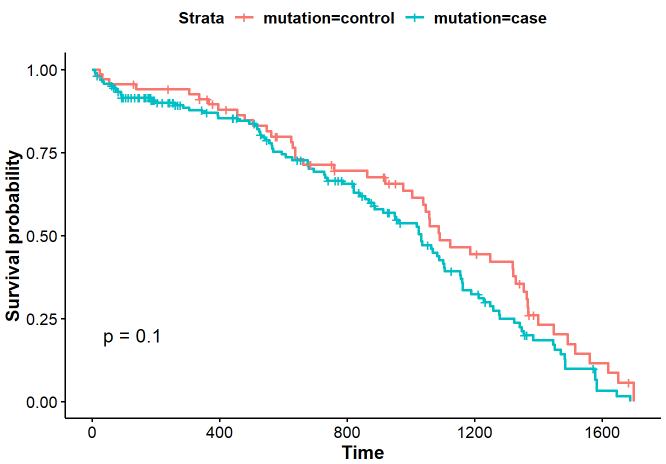


**Figure S4.** Comparison of Kaplan Meier curves of the overall survival between mutation carrier group (case) and mutation non-carrier group (control) in OV. The p-value shown is from the log-rank test.


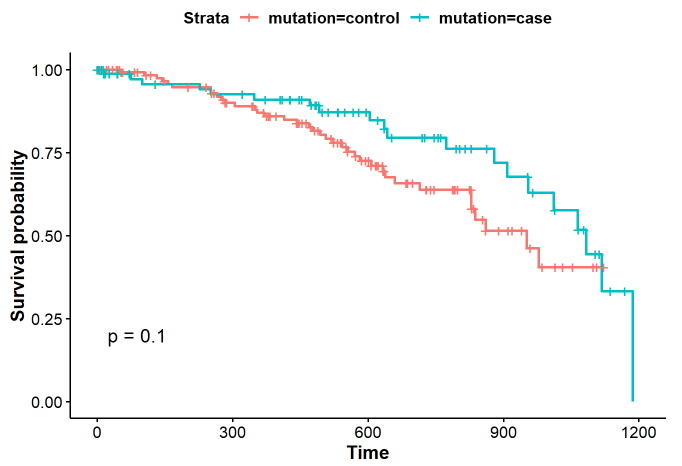


**Figure S5.** Comparison of Kaplan Meier curves of the overall survival between mutation carrier group (case) and mutation non-carrier group (control) in CESC. The p-value shown is from the log-rank test.


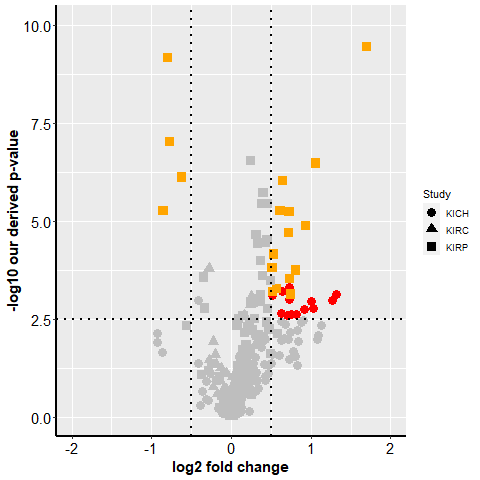


**Figure S6.** Volcano plot of miRNAs with |$w_{g}^{*}|=(1,0,1)$ (corresponding to KICH, KIRC and KIRP) for Pan-Kidney cohort. The miRNAs from KICH, KIRC and KIRP are shaped as circle, triangle and square, respectively. The miRNAs having |LFC| > 0.5 (dotted line) and -log10(p) > 2.5 (dotted line) are highlighted (red and orange for KICH and KIRP, respectively). The other miRNAs are colored in grey.

**
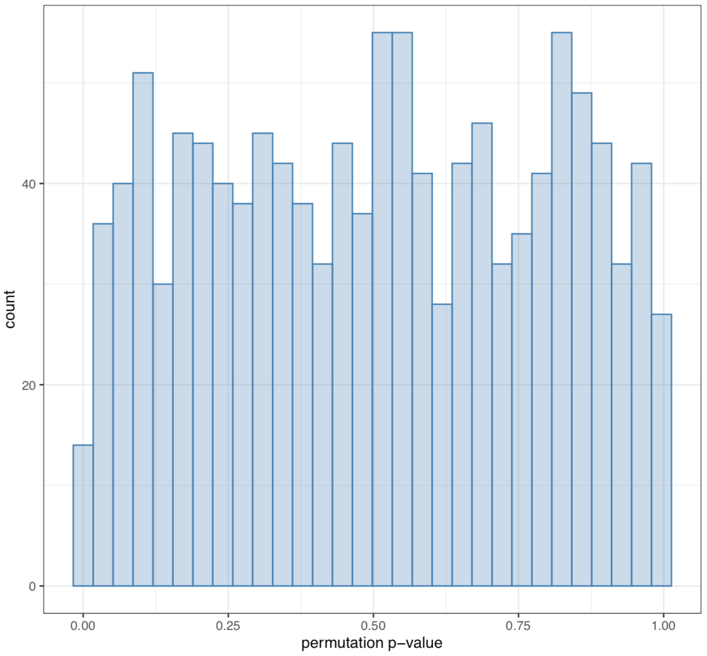
**

**Figure S7.** Distribution of permutated p-value for dominant pattern using the null genes in the simulation.


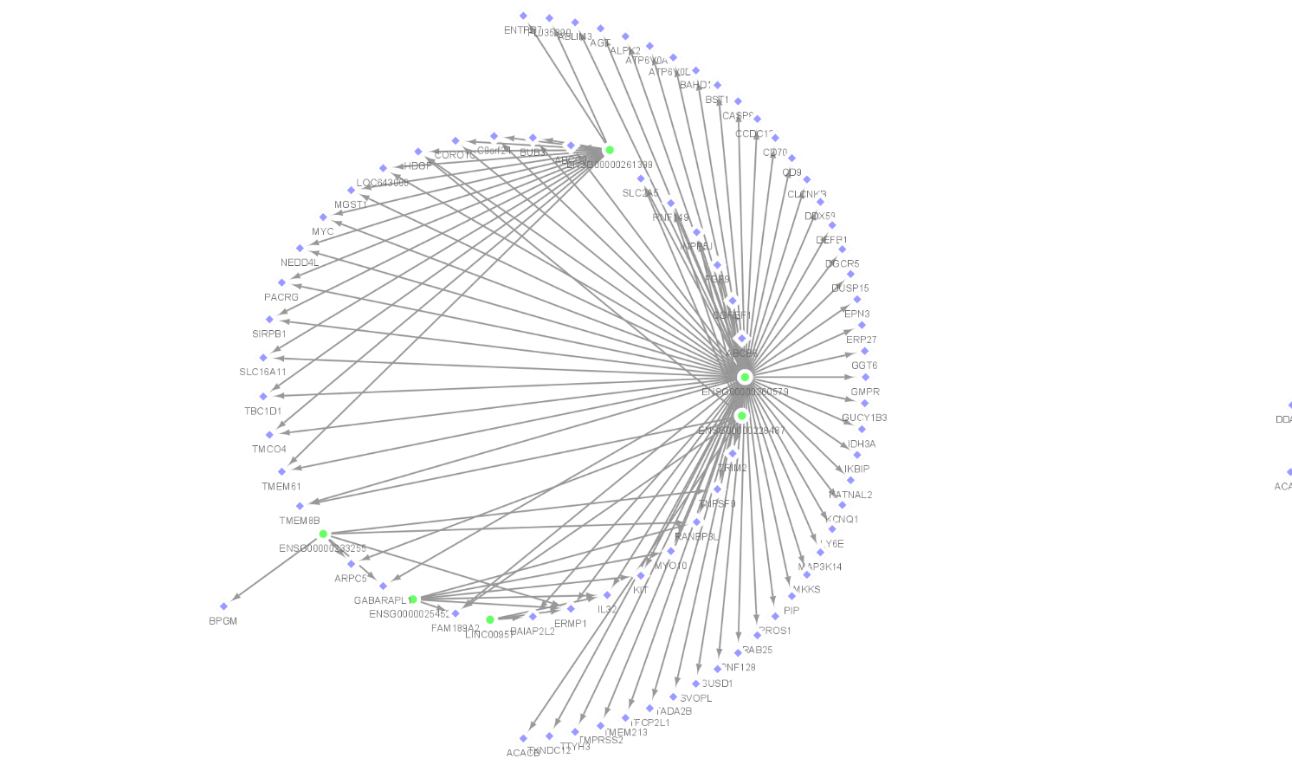

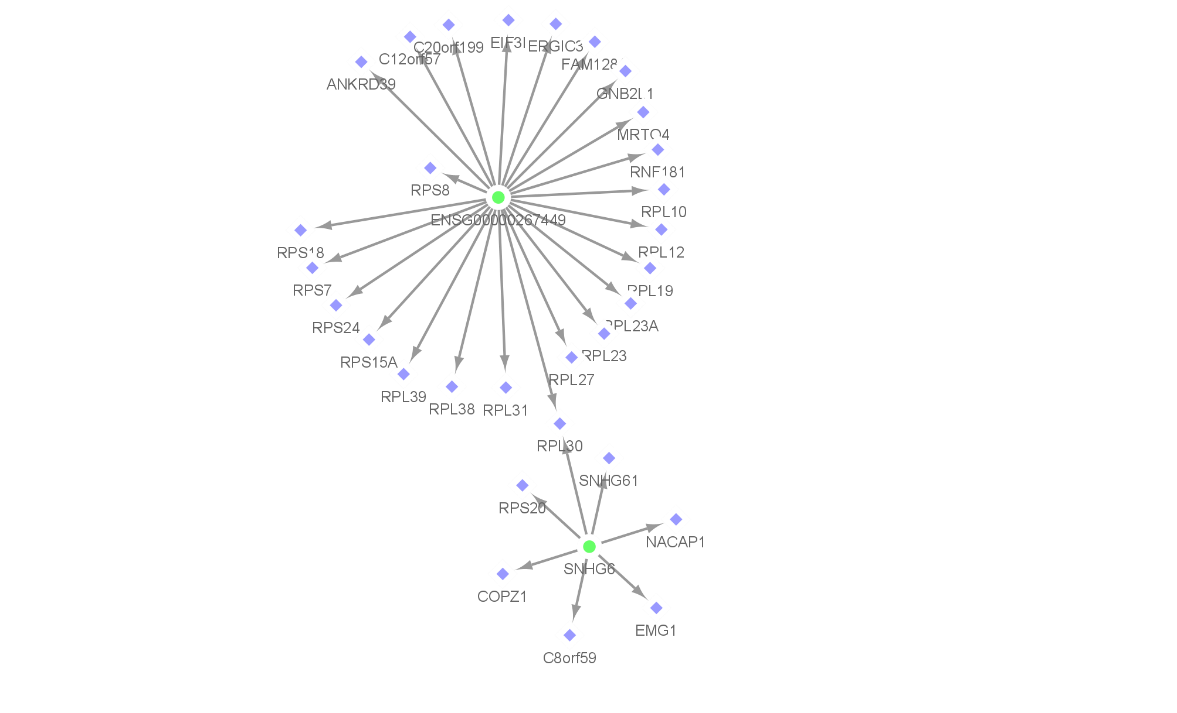


**Figure S8.** Two lncRNA-mRNA regulatory network identified from biomarkers with |$w_{g}^{*}|=$ (1,1,1) (corresponding to KICH, KIRC and KIRP, respectively) for the Pan-Kidney example. The circle shapes represent lncRNAs highlighted in green and diamond shapes represent mRNAs highlighted in purple. The arrows indicate the network relationships between lncRNAs and mRNAs.

**Bibliography**

1. An, H., et al., *Up-regulation of long non-coding RNA SNHG6 predicts poor prognosis in renal cell carcinoma.* Eur Rev Med Pharmaco, 2018. **24**(22): p. 8624-8629.

2. Wu, G., et al., *A new survival model based on ferroptosis-related genes for prognostic prediction in clear cell renal cell carcinoma.* Aging (Albany NY), 2020. **12**(14): p. 14933.

3. Zhou, X., et al., *Ribosomal proteins: functions beyond the ribosome.* Journal of molecular cell biology, 2015. **7**(2): p. 92-104.

4. Bryzghalov, O., M.W. Szcześniak, and I. Makałowska, *Retroposition as a source of antisense long non-coding RNAs with possible regulatory functions.* Acta Biochimica Polonica, 2016. **63**(4): p. 825-833.

5. Guo, H., et al., *The PI3K/AKT pathway and renal cell carcinoma.* Journal of genetics and genomics, 2015. **42**(7): p. 343-353.

6. Tan, J.-Y., et al., *Screening and verification of proteins that interact with HSPC238.* Oncology reports, 2015. **34**(6): p. 3097-3103.

7. Dolezal, J.M., A.P. Dash, and E.V. Prochownik, *Diagnostic and prognostic implications of ribosomal protein transcript expression patterns in human cancers.* BMC cancer, 2018. **18**(1): p. 275.

8. Stelzer, G., et al., *The GeneCards suite: from gene data mining to disease genome sequence analyses.* Current protocols in bioinformatics, 2016. **54**(1): p. 1.30. 1-1.30. 33.

9. Zhong, W., et al., *Identification of Epithelial-Mesenchymal Transition-Related lncRNA With Prognosis and Molecular Subtypes in Clear Cell Renal Cell Carcinoma.* Oncol, 2020. **10**: p. 591254.

10. Perron, U., P. Provero, and I. Molineris, *In silico prediction of lncRNA function using tissue specific and evolutionary conserved expression.* Bmc Bioinformatics, 2017. **18**(5): p. 29-39.

11. Yates, A.D., et al., *Ensembl 2020.* Nucleic acids research, 2020. **48**(D1): p. D682-D688.

12. Xiao, W., et al., *TRIM2 downregulation in clear cell renal cell carcinoma affects cell proliferation, migration, and invasion and predicts poor patients’ survival.* Cancer management and research, 2018. **10**: p. 5951.

13. Anandasabapathy, N., et al., *GRAIL: an E3 ubiquitin ligase that inhibits cytokine gene transcription is expressed in anergic CD4+ T cells.* Immunity, 2003. **18**(4): p. 535-547.

14. Jin, X., et al., *RNF13: an emerging RING finger ubiquitin ligase important in cell proliferation.* The FEBS journal, 2011. **278**(1): p. 78-84.

15. Sun, C.-Y., et al., *A novel SNP in the 5’regulatory region of organic anion transporter 1 is associated with chronic kidney disease.* Scientific reports, 2018. **8**(1): p. 1-10.

16. Toma, M.I., et al., *PARK2 and PACRG are commonly downregulated in clear‐cell renal cell carcinoma and are associated with aggressive disease and poor clinical outcome.* Genes, Chromosomes and Cancer, 2013. **52**(3): p. 265-273.
